# Supplementary material for: Hot spot prediction in protein-protein interactions by an ensemble system
Source: BMC Syst Biol. 2018 Dec 31;12(Suppl 9):132. doi: 10.1186/s12918-018-0665-8 (PMC6311905; doi:10.1186/s12918-018-0665-8)
Supplement: Supplementary file 2 — All relevant MATLAB code. A simple Matlab implement of our predictor. (ZIP 19,742 kb) [file 12918_2018_665_MOESM2_ESM.zip › Additional file 2/Read ASA/ASA website/dbMPIKT/4JGHA.html]

 NetSurfP - Protein Surface Accessibility and Secondary Structure Predictions

  

|  |  |  |
| --- | --- | --- |
|  |  | NetSurfP - Protein Surface Accessibility and Secondary Structure Predictions  Technical University of Denmark |

  


---

```
# For publication of results, please cite:
# A generic method for assignment of reliability scores applied to solvent accessibility predictions.
# Bent Petersen, Thomas Nordahl Petersen, Pernille Andersen, Morten Nielsen and Claus Lundegaard
# BMC Structural Biology 2009, 9:51 doi:10.1186/1472-6807-9-51
#
# Column 1: Class assignment - B for buried or E for Exposed - Threshold: 25% exposure, but not based on RSA
# Column 2: Amino acid
# Column 3: Sequence name
# Column 4: Amino acid number
# Column 5: Relative Surface Accessibility - RSA
# Column 6: Absolute Surface Accessibility
# Column 7: Z-fit score for RSA prediction
# Column 8: Probability for Alpha-Helix
# Column 9: Probability for Beta-strand
# Column 10: Probability for Coil
E H  4JGHA                  1    0.904 164.510  -0.877   0.003   0.003   0.994
E M  4JGHA                  2    0.430  86.123  -1.409   0.005   0.015   0.979
E D  4JGHA                  3    0.641  92.354   0.799   0.005   0.015   0.979
E P  4JGHA                  4    0.310  44.032  -1.591   0.053   0.005   0.942
E E  4JGHA                  5    0.698 121.958  -0.209   0.321   0.003   0.675
E F  4JGHA                  6    0.618 123.952  -0.523   0.406   0.004   0.590
E Q  4JGHA                  7    0.458  81.727  -0.450   0.339   0.016   0.645
E A  4JGHA                  8    0.327  36.013  -1.136   0.354   0.048   0.598
E A  4JGHA                  9    0.411  45.259  -0.820   0.184   0.043   0.773
E R  4JGHA                 10    0.543 124.416  -0.260   0.184   0.043   0.773
B L  4JGHA                 11    0.153  27.941   0.220   0.184   0.043   0.773
E A  4JGHA                 12    0.279  30.757   0.061   0.268   0.043   0.689
E K  4JGHA                 13    0.677 139.177   0.438   0.430   0.016   0.555
E A  4JGHA                 14    0.511  56.279   0.494   0.522   0.016   0.462
E L  4JGHA                 15    0.276  50.591  -0.239   0.622   0.015   0.363
E R  4JGHA                 16    0.588 134.767   0.806   0.622   0.015   0.363
E E  4JGHA                 17    0.549  95.840   1.451   0.622   0.015   0.363
B L  4JGHA                 18    0.079  14.520   0.040   0.622   0.015   0.363
E G  4JGHA                 19    0.496  39.043   0.618   0.522   0.016   0.462
E Q  4JGHA                 20    0.732 130.807   0.408   0.257   0.016   0.727
B T  4JGHA                 21    0.232  32.123   1.030   0.058   0.017   0.925
E G  4JGHA                 22    0.441  34.675  -0.438   0.053   0.043   0.903
B W  4JGHA                 23    0.096  23.184   0.263   0.064   0.216   0.721
B Y  4JGHA                 24    0.125  26.670  -0.031   0.066   0.296   0.638
B W  4JGHA                 25    0.269  64.670   0.224   0.066   0.296   0.638
E G  4JGHA                 26    0.331  26.026  -0.737   0.019   0.141   0.840
E S  4JGHA                 27    0.602  70.519  -0.212   0.018   0.088   0.893
B M  4JGHA                 28    0.070  13.927   0.241   0.005   0.045   0.951
E T  4JGHA                 29    0.451  62.526   1.074   0.005   0.015   0.979
B V  4JGHA                 30    0.208  31.970   0.673   0.923   0.002   0.076
E N  4JGHA                 31    0.631  92.320   1.270   0.970   0.001   0.030
E E  4JGHA                 32    0.424  73.985   1.726   0.970   0.001   0.030
B A  4JGHA                 33    0.018   1.995   1.021   0.970   0.001   0.030
E K  4JGHA                 34    0.256  52.597   1.166   0.970   0.001   0.030
E E  4JGHA                 35    0.570  99.579   1.552   0.970   0.001   0.030
B K  4JGHA                 36    0.196  40.420   1.638   0.923   0.002   0.076
B L  4JGHA                 37    0.033   6.006  -0.634   0.858   0.002   0.139
E K  4JGHA                 38    0.469  96.432   1.037   0.694   0.003   0.303
E E  4JGHA                 39    0.742 129.662  -0.444   0.176   0.004   0.820
E A  4JGHA                 40    0.331  36.443   0.076   0.016   0.005   0.979
E P  4JGHA                 41    0.577  81.820  -1.734   0.005   0.015   0.979
E E  4JGHA                 42    0.415  72.466  -0.320   0.005   0.045   0.951
B G  4JGHA                 43    0.040   3.148  -0.961   0.005   0.262   0.733
B T  4JGHA                 44    0.076  10.541  -0.099   0.003   0.718   0.279
B F  4JGHA                 45    0.030   6.101   0.309   0.001   0.900   0.099
B L  4JGHA                 46    0.020   3.735   1.192   0.001   0.900   0.099
B I  4JGHA                 47    0.019   3.496   1.387   0.001   0.900   0.099
B R  4JGHA                 48    0.088  20.106   0.569   0.002   0.816   0.182
B D  4JGHA                 49    0.184  26.486   0.074   0.004   0.514   0.481
B S  4JGHA                 50    0.082   9.599  -0.840   0.004   0.197   0.799
E S  4JGHA                 51    0.584  68.410  -0.806   0.004   0.085   0.910
E H  4JGHA                 52    0.401  72.978  -0.490   0.005   0.045   0.951
E S  4JGHA                 53    0.544  63.769  -0.753   0.005   0.015   0.979
E D  4JGHA                 54    0.586  84.399  -0.804   0.005   0.015   0.979
B Y  4JGHA                 55    0.286  61.075  -1.108   0.004   0.197   0.799
B L  4JGHA                 56    0.228  41.820  -0.095   0.004   0.514   0.481
B L  4JGHA                 57    0.052   9.594   0.622   0.001   0.900   0.099
B T  4JGHA                 58    0.022   2.982   0.502   0.001   0.959   0.040
B I  4JGHA                 59    0.015   2.756   1.636   0.001   0.959   0.040
B S  4JGHA                 60    0.024   2.825   0.323   0.001   0.959   0.040
B V  4JGHA                 61    0.024   3.658   0.816   0.001   0.959   0.040
B K  4JGHA                 62    0.157  32.315   0.219   0.001   0.959   0.040
B T  4JGHA                 63    0.123  17.074  -0.247   0.001   0.900   0.099
E S  4JGHA                 64    0.663  77.692  -0.134   0.005   0.045   0.951
E A  4JGHA                 65    0.755  83.245  -1.430   0.003   0.003   0.994
E G  4JGHA                 66    0.447  35.171   0.682   0.004   0.420   0.576
B P  4JGHA                 67    0.053   7.535  -0.342   0.002   0.816   0.182
B T  4JGHA                 68    0.180  25.008   1.863   0.001   0.900   0.099
B N  4JGHA                 69    0.062   9.048   0.011   0.001   0.900   0.099
B L  4JGHA                 70    0.041   7.489   0.841   0.001   0.900   0.099
B R  4JGHA                 71    0.124  28.442   1.377   0.001   0.959   0.040
B I  4JGHA                 72    0.018   3.404   1.141   0.001   0.959   0.040
E E  4JGHA                 73    0.250  43.640   1.163   0.001   0.900   0.099
B Y  4JGHA                 74    0.212  45.262   0.456   0.002   0.816   0.182
E Q  4JGHA                 75    0.540  96.426  -0.019   0.004   0.420   0.576
E D  4JGHA                 76    0.779 112.254  -1.208   0.005   0.015   0.979
E G  4JGHA                 77    0.612  48.141  -1.697   0.005   0.045   0.951
E K  4JGHA                 78    0.400  82.342  -0.053   0.004   0.616   0.381
B F  4JGHA                 79    0.142  28.479  -0.193   0.002   0.816   0.182
E R  4JGHA                 80    0.286  65.425  -0.172   0.002   0.816   0.182
B L  4JGHA                 81    0.112  20.452  -1.551   0.003   0.718   0.279
E D  4JGHA                 82    0.428  61.660  -1.345   0.005   0.336   0.660
E S  4JGHA                 83    0.626  73.402  -2.588   0.018   0.088   0.893
E I  4JGHA                 84    0.445  82.233  -1.226   0.020   0.205   0.775
E I  4JGHA                 85    0.336  62.160  -1.179   0.022   0.552   0.426
E C  4JGHA                 86    0.307  43.117  -0.643   0.023   0.655   0.322
B V  4JGHA                 87    0.230  35.382  -1.402   0.022   0.552   0.426
E K  4JGHA                 88    0.432  88.862  -0.625   0.021   0.451   0.528
E S  4JGHA                 89    0.412  48.251  -1.402   0.021   0.279   0.699
E K  4JGHA                 90    0.588 121.013  -1.136   0.019   0.141   0.840
E L  4JGHA                 91    0.591 108.139  -0.400   0.018   0.088   0.893
E K  4JGHA                 92    0.416  85.530   0.250   0.018   0.088   0.893
E Q  4JGHA                 93    0.511  91.247   0.729   0.019   0.141   0.840
B F  4JGHA                 94    0.041   8.168  -0.774   0.018   0.088   0.893
E D  4JGHA                 95    0.606  87.368   0.491   0.018   0.019   0.964
B S  4JGHA                 96    0.168  19.748  -0.363   0.115   0.016   0.868
B V  4JGHA                 97    0.080  12.219   0.299   0.970   0.001   0.030
B V  4JGHA                 98    0.261  40.039   0.811   0.970   0.001   0.030
E H  4JGHA                 99    0.419  76.162   1.315   0.988   0.000   0.012
B L  4JGHA                100    0.029   5.292   0.696   0.988   0.000   0.012
B I  4JGHA                101    0.038   6.938  -0.229   0.988   0.000   0.012
E D  4JGHA                102    0.404  58.216   1.198   0.988   0.000   0.012
B Y  4JGHA                103    0.226  48.296   1.131   0.970   0.001   0.030
B Y  4JGHA                104    0.043   9.275   0.312   0.970   0.001   0.030
E V  4JGHA                105    0.283  43.559   0.528   0.938   0.007   0.055
E Q  4JGHA                106    0.588 104.999   0.348   0.879   0.010   0.111
E M  4JGHA                107    0.366  73.237  -0.307   0.502   0.002   0.495
B C  4JGHA                108    0.314  44.100  -1.410   0.246   0.004   0.750
E K  4JGHA                109    0.488 100.279  -1.136   0.181   0.016   0.803
E D  4JGHA                110    0.511  73.621  -1.887   0.115   0.016   0.868
E K  4JGHA                111    0.419  86.188  -1.699   0.113   0.043   0.844
E R  4JGHA                112    0.469 107.355  -1.620   0.113   0.043   0.844
E T  4JGHA                113    0.435  60.376  -1.311   0.113   0.087   0.800
E G  4JGHA                114    0.459  36.115  -1.202   0.056   0.142   0.802
E P  4JGHA                115    0.359  50.956  -1.735   0.056   0.142   0.802
E E  4JGHA                116    0.508  88.817  -1.148   0.056   0.142   0.802
E A  4JGHA                117    0.323  35.617  -0.847   0.052   0.084   0.864
E P  4JGHA                118    0.396  56.164  -1.565   0.052   0.084   0.864
E R  4JGHA                119    0.347  79.532  -1.378   0.052   0.084   0.864
E N  4JGHA                120    0.662  96.858  -1.741   0.018   0.088   0.893
E G  4JGHA                121    0.426  33.550  -1.066   0.018   0.088   0.893
E T  4JGHA                122    0.531  73.622  -1.034   0.019   0.141   0.840
E V  4JGHA                123    0.406  62.418  -0.957   0.019   0.141   0.840
E H  4JGHA                124    0.441  80.163  -0.910   0.021   0.279   0.699
B L  4JGHA                125    0.145  26.623  -1.352   0.021   0.451   0.528
E Y  4JGHA                126    0.378  80.864   0.354   0.022   0.552   0.426
B L  4JGHA                127    0.107  19.628  -1.190   0.021   0.451   0.528
E T  4JGHA                128    0.438  60.764  -1.125   0.022   0.359   0.619
E K  4JGHA                129    0.328  67.511  -0.382   0.019   0.141   0.840
B P  4JGHA                130    0.239  33.957  -1.776   0.018   0.047   0.935
B L  4JGHA                131    0.189  34.551  -0.573   0.005   0.015   0.979
E Y  4JGHA                132    0.427  91.229  -0.878   0.005   0.015   0.979
E T  4JGHA                133    0.491  68.088  -1.172   0.005   0.015   0.979
E S  4JGHA                134    0.624  73.074  -1.657   0.003   0.003   0.994
E A  4JGHA                135    0.465  51.210  -0.794   0.053   0.005   0.942
E P  4JGHA                136    0.366  51.879  -0.168   0.321   0.003   0.675
E S  4JGHA                137    0.377  44.173   0.334   0.694   0.003   0.303
B L  4JGHA                138    0.117  21.404  -1.000   0.858   0.002   0.139
E Q  4JGHA                139    0.296  52.830   0.147   0.802   0.014   0.185
E H  4JGHA                140    0.291  52.969  -0.022   0.802   0.014   0.185
B L  4JGHA                141    0.118  21.606  -0.372   0.802   0.014   0.185
B C  4JGHA                142    0.058   8.087   0.655   0.660   0.049   0.291
E R  4JGHA                143    0.266  60.868   1.004   0.751   0.050   0.199
E L  4JGHA                144    0.311  56.926   0.236   0.779   0.100   0.120
B T  4JGHA                145    0.141  19.515   0.754   0.807   0.137   0.056
B I  4JGHA                146    0.040   7.326  -0.252   0.779   0.100   0.120
E N  4JGHA                147    0.325  47.536   0.959   0.779   0.100   0.120
E K  4JGHA                148    0.568 116.838   0.718   0.701   0.107   0.192
B C  4JGHA                149    0.253  35.493   0.228   0.605   0.105   0.290
E T  4JGHA                150    0.341  47.338  -0.408   0.268   0.043   0.689
E G  4JGHA                151    0.594  46.756  -1.138   0.113   0.043   0.844
E A  4JGHA                152    0.507  55.915   0.092   0.058   0.017   0.925
E I  4JGHA                153    0.310  57.405  -0.191   0.113   0.043   0.844
E W  4JGHA                154    0.622 149.519  -0.776   0.053   0.043   0.903
E G  4JGHA                155    0.561  44.174   0.147   0.053   0.043   0.903
B L  4JGHA                156    0.158  28.966   0.091   0.053   0.043   0.903
E P  4JGHA                157    0.528  74.980  -0.563   0.053   0.043   0.903
B L  4JGHA                158    0.131  24.041   0.347   0.058   0.017   0.925
B P  4JGHA                159    0.211  29.884   0.153   0.181   0.016   0.803
E T  4JGHA                160    0.560  77.630   0.767   0.782   0.003   0.216
E R  4JGHA                161    0.421  96.363   1.616   0.782   0.003   0.216
B L  4JGHA                162    0.030   5.438   0.166   0.923   0.002   0.076
E K  4JGHA                163    0.294  60.496   1.389   0.970   0.001   0.030
E D  4JGHA                164    0.529  76.229   1.400   0.970   0.001   0.030
B Y  4JGHA                165    0.095  20.216   1.225   0.923   0.002   0.076
B L  4JGHA                166    0.063  11.480   0.537   0.858   0.002   0.139
E E  4JGHA                167    0.616 107.598   0.748   0.782   0.003   0.216
E E  4JGHA                168    0.684 119.407   1.172   0.502   0.002   0.495
E Y  4JGHA                169    0.278  59.344   0.960   0.109   0.005   0.886
E K  4JGHA                170    0.565 116.159   0.209   0.018   0.019   0.964
B F  4JGHA                171    0.255  51.138  -0.116   0.018   0.088   0.893
E Q  4JGHA                172    0.566 101.070   0.936   0.019   0.141   0.840
E V  4JGHA                173    0.550  84.535  -1.479   0.003   0.003   0.994

Explain the output.  Go back.
```

---
